# Fragmented QRS and prediction of paroxysmal atrial fibrillation episodes

**Published:** 2014

**Authors:** Ahmet Temiz, Emine Gazi, Ömer Güngör, Burak Altun, Ahmet Barutcu, Adem Bekler, Yusuf Ziya Tan, Sedat Ozcan, Ali Ümit Yener, Tolga Kurt

**Affiliations:** 1 Ahmet Temiz, MD, Assistant Professor of Cardiology, Çanakkale 18 Mart University Medical Faculty, Çanakkale, Turkey.; 2 Emine Gazi, MD, Assistant Professor of Cardiology, Çanakkale 18 Mart University Medical Faculty, Çanakkale, Turkey.; 3 Ömer Güngör, MD, Specialist in Cardiology, Department of Cardiology,Çanakkale State Hospital, Çanakkale, Turkey.; 4 Burak Altun, MD, Assistant Professor of Cardiology, Çanakkale 18 Mart University Medical Faculty, Çanakkale, Turkey.; 5 Ahmet Barutcu, MD, Assistant Professor of Cardiology, Çanakkale 18 Mart University Medical Faculty, Çanakkale, Turkey.; 6 Adem Bekler, MD, Assistant Professor of Cardiology, Çanakkale 18 Mart University Medical Faculty, Çanakkale, Turkey.; 7 Yusuf Ziya Tan, MD, Assistant Professor of Nuclear Medicine, Çanakkale 18 Mart University Medical Faculty, Çanakkale, Turkey.; 8 Sedat Ozcan, MD, Assistant Professor of Cardiovascular Surgery, Çanakkale 18 Mart University Medical Faculty, Çanakkale, Turkey.; 9 Ali Ümit Yener, MD, Assistant Professor of Cardiovascular Surgery, Çanakkale 18 Mart University Medical Faculty, Çanakkale, Turkey.; 10 Tolga Kurt, MD, Assistant Professor of Cardiovascular Surgery, Çanakkale 18 Mart University Medical Faculty, Çanakkale, Turkey.

**Keywords:** Fragmented QRS, Paroxysmal atrial fibrillation, Holter monitoring

## Abstract

***Objective:*** Prior studies have demonstrated the relationship between cardiovascular diseases and fragmented QRS (fQRS). fQRS was also associated with ventricular arrhythmias. Our objective was to find out the relationship between fQRS and paroxysmal atrial fibrillation (PAF).

***Method:*** A total of 301 patients without overt structural heart disease were prospectively included in the study. Patients were divided in to 2 groups according to presence of fQRS. Multivariate logistic regression analysis was used to assess the predictive value of fQRS for predicting PAF.

***Results:*** One hundred and three patients had fQRS. Patients with fQRS were older (53±16.8 vs 45.3±17.2, p<0.001), with larger left atrium (LA) (33.2±5.9 vs 30.1±5.9 mm, p=0.001), with thicker interventricular septum (IVS) (10.2±1.9 vs 9.5±2.3 mm, p=0.032), more diabetic (19.8 vs 10.6%, p=0.029) and have more PAF episodes (22.3 vs 4.1%, p<0.001) in comparison with patients without fQRS. fQRS was an independent predictor of detecting PAF episode (odds ratio, 9.69; 95% confidence interval, 2.46-38.15, p=0.001). Hypertension and diabetes mellitus were also predictive.

***Conclusion:*** The presence of fQRS independently predicted PAF episodes in holter monitoring (HM). Further studies are needed to clarify the clinical implications of this finding.

## INTRODUCTION

The term fragmented QRS (fQRS) represents the various RSR’ patterns (≥1 R’ or notching of S wave or R wave) in two contiguous leads corresponding to a major coronary artery territory.^1^ fQRS is a predictor of cardiovascular events and/or mortality in patients with a spectrum of cardiovascular diseases including acute coronary syndrome (ACS), dilated cardiomyopathy and heart failure.^2^^-^^5^ Furthermore fQRS was found to be associated with lethal ventricular arrhythmias and sudden cardiac death.^5^^,^^6^

The reason for all the above mentioned associations between fQRS and various cardiovascular diseases was found to be cardiac fibrosis.^7^ Additionally, heterogeneous activity of ischemic ventricle alters the ventricle depolarization and may be responsible for fQRS formation.^8^ It is reasonable to anticipate that these changes in the ventricle may influence the atrium and cause atrial arrhythmias. Indeed it was demonstrated that the fQRS was as predictor of the occurrence of post-operative atrial fibrillation (AF) in isolated coronary artery by-pass surgery.^9^

We hypothesized that presence of fQRS may be useful for predicting atrial arrhythmias, especially paroxysmal AF (PAF) which is the most common cardiac arrhythmia with important clinical implications.

## METHODS

***Study design: ***This was a prospective observational study.

***Study population: ***A total of 301 patients, who were referred for 24 h Holter ECG monitoring because of complaint of palpitation, at our institution between June 2012 and December 2012 were enrolled consecutively.

Pediatric Holters, pacemaker-dependent patients, patients with complete bundle branch block and patients with ongoing antiarrhythmic therapy, patients with thyroid disorders, patients with severe cardiac diseases including valvular disease, heart failure and cardiomyopathies were excluded. We also excluded the Holters which were performed to hospitalized patients The study was carried out in accordance with Declaration of Helsinki principles and was approved by the Local Ethics Committee.

***Study protocol: ***The patients referred for 24 h Holter ECG monitoring were evaluated by a cardiologist before holter evaluation. Each patient’s history and examination was collected by a cardiologist. We recorded the baseline characteristics, which included hypertension (HT), smoking status, diabetes mellitus (DM), family history of CAD, height and weight and medication. 

Routine laboratory measurement, which were obtained after at least 8 h fasting, including glucose, creatinine, lipid profile, thyroid stimulating hormone (TSH) and hemogram were recorded.

Basic echocardiography measurements including chamber diameters, wall thicknesses an ejection fraction(EF) obtained with Vivid 7 (GE Vingmed Ultrasound AS, Horten, Norway) according to the guidelines of the American Society of Echocardiography^10^ and recorded on echopacs.

***Electrocardiography: ***A 12 lead surface ECG was obtained from all patients before connecting the Holter device to the patient. This 12- lead ECGs (Nihon-KohdenCardiofax ECG1350K, Tokyo, Japan, filter range 0.5 Hz to 150 Hz, AC filter 60 Hz, 25 mm/s, 10 mm/mV) were analyzed by 2 independent cardiologist who were blinded to Holter data. fQRS was defined as presence of different RSR’ patterns (QRS duration <120 ms) which include an additional R wave (R’ prime) or notching of the R wave or S wave, or the presence of more than one R’ prime without typical bundle branch block in two contiguous leads corresponding to a major coronary artery territory. The inter-observer concordance rate for determining fQRS was 98.2% between two readers. In case of disagreement, the final decision was made mutually. We also evaluated the number of fQRS, because fragmentation in only one lead is not accepted as the presence of fQRS; we accepted as absence of fQRS if the number of fQRS was zero or one.

***Holter monitoring and interpretation: ***Then two independent cardiologist evaluated the recordings for presence of any AF episodes. More than 3 consecutive QRS complexes without P wave and with irregular RR intervals was accepted as PAF. The inter-observer concordance rate for determining AF episodes was 99.7% between two readers. If the device software defined AF or supraventricular arrhythmia these tracings *were* evaluated visually to confirm the presence of AF episodes.

***Statistical analysis: ***All statistical studies were carried out with the program SPPS (version 15.0, SPSS, Chicago, Illinois, USA). Quantitative variables were expressed as the mean value ±SD or median (minimum-maximum), and qualitative variables were expressed as percentages (%). All measurements were evaluated with the Kolmogorov-Smirnov test. A comparison of parametric values between two groups, according to presence of fQRS, was performed using the Mann-Whitney U-test or student t test. The study population was divided into three groups based on fQRS number. Groups 1, 2 and 3 were defined as the presence of fQRS in 0 or 1 lead, in 2 or 3 leads, and in ≥4 leads in electrocardiogram, respectively. A comparison between these groups was performed using Kruskal-Wallis test and if there was significance, a Mann-Whitney U test was used for post hoc analysis. Categorical variables were compared by the likelihood-ratio *χ*^2^ test or Fisher’s exact test. 

A backward stepwise multivariate logistic regression analysis which included variables with p < 0.1 was performed to identify independent predictors of AF episodes. Age ≥65, increase left atrium diameter (≥35 mm), increase interventricular septum diameter (≥11 mm), male gender, DM, HT, family history, beta-blocker treatment using and presence of fQRS were entered into the model. A p <0.05 (***two-sided)*** was considered significant. 

## RESULTS

A total of 301 patients included in the study. fQRS was present in 103 patients. PAF episodes were seen in 31 patients. Baseline clinical characteristics are presented in Table-I. Patients with fQRS were older (p<0.001), with larger left atrium (LA) (p=0.001), with thicker interventricular septum (IVS) (p=0.032), more had DM and AF episodes (p<0.001) in comparison with patients without fQRS. Differences with respect to number of fQRS lead are detailed in Table-II, while group 1 describing patient with 0 or 1 lead, group 2 with 2 or 3 leads and group 3 with > 3 leads with fQRS. Presence of AF episodes (p<0.001), age (p=0.001), beta-blocker use (p=0.016), fasting blood glucose levels (p=0.015), TSH levels (p=0.049) were found to be increased with raised numbers of fQRS. Table-III presents the dependent parameters for predicting AF episodes and Table-IV shows the independent predictors of AF episodes. Presence of fQRS, HT and DM were independent predictors of AF episodes in 24 h HM.

**Fig.1:**
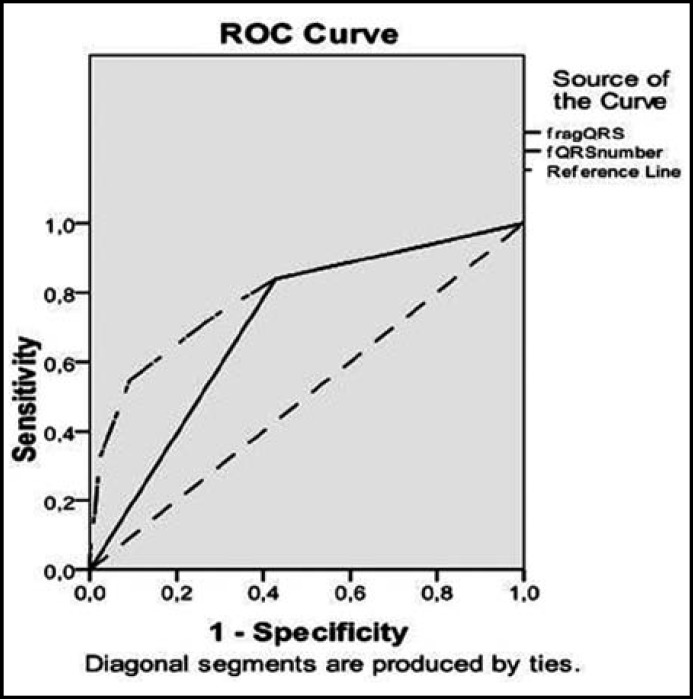
The sensitivity and specificity of fQRS and number of fQRS for detecting PAF episodes in HM

The area under the receiver operating characteristics (ROC) curve values for the presence of fQRS and number of fQRS to detect PAF on HM were 0.704 and 0.797 (Fig.1). Presence of fQRS had 74% sensivity and 71% specificity for detecting PAF, the specificity was increased with the increased number of fQRS leads (91% for 3 leads and 98% for 4 leads) while the sensivity was decreased (54% for 3 leads, 32% for 4 leads).

## DISCUSSION

The main finding of our study is that presence of fQRS on 12 lead surfaces ECG is related with presence of PAF episodes in 24 h ambulatory HM. Additionally, increasing number of fQRS leads were also associated with increased presence of PAF episodes. To our knowledge this is the first study that demonstrates the relationship between fQRS and presence of PAF episodes in 24 h ECG monitoring.

AF is most common cardiac rhythm disorder and it significantly increases mortality and morbidity.^11^ Mean catastrophic consequences of AF is stroke; the risk of stroke is 5 times higher in patient with non valvular AF than the general population.^12^ Because of these important clinical implications of AF, timely diagnosis and management is mandatory. The problem for diagnosing AF is that, it is not always symptomatic and not always detectable during examination or surface ECG due to its transient episodic nature. To detect PAF extended ECG monitoring is usually needed. Thirty seconds cut off for duration of PAF is used generally since it was first mentioned in the 2006 American Heart Association atrial fibrillation guidelines but this has been questioned nowadays, because it has been suggested that PAF episodes shorter than 30 sec may be indicator of longer episodes.^13^ These short episodes may become longer and finally may persist over time.^14^ Additionally, premature atrial contractions (PACs) detected by HM were found to be associated with recurrence of AF.^15^ In addition to PACs, other silent brief atrial tachyarrhythmias may result in increased stroke risk and finally may convert to clinically certain AF.^16^ As a consequence, it will not be wrong if we suggest that any brief atrial arrhythmia may predict future AF occurrences. Indeed longer rhythm monitoring showed that substantial proportion of patients, who were initially diagnosed as cryptogenic stroke, have AF episodes.^17^

**Table-I:** Baseline characteristics of the study population

	***fQRS (-) (n=198)***	***fQRS (+) (n=103)***	***p***
Gender (male) (%)	36.9 (73)	48.5 (50)	0.051
Age (years)	45.3±17.2	53.5±16.8	<0.001
BMI (kg/m^2^)	27.4±5.1	26,8±6.1	0.382
Hypertension (%)	28.3 (56)	37.3 (38)	0.113
Diabetes mellitus (%)	10.6 (21)	19.8 (20)	0.029
Smoking (%)	25.3 (50)	22.5 (23)	0.605
Family history of CAD (%)	12.1 (24)	23.8 (24)	0.010
Previus MI (%)	2 (4)	2.9 (3)	0.693
Ejection fraction (%)	63.4±4.1	61.9±6.5	0.056
LVEDD (mm)	42.6±4.6	43.9±4.9	0.082
LVESD (mm)	28.1±4.9	28.5±4.2	0.499
PWd (mm)	9.7±1.9	10.2±2.4	0.096
IVSd (mm)	9.5±2.3	10.2±1.9	0.032
LA (mm)	30.1±5.9	33.2±5.9	0.001
BB (%)	15.7 (31)	29.1 (30)	0.006
ND-CCB (%)	8.7 (17)	14.6 (15)	0.117
FPG (mg/dL)	91 (70-196)	96.5 (73-359)	0.007
Creatinin (mg/dL)	0.70(0.47-4.15)	0.75 (0.48-2.69)	0.665
Total cholesterol(mg/dL)	200.1±37.3	223.6±91.8	0.078
Triglyceride (mg/dL)	92 (28-283)	113.5 (11-368)	0.063
LDL-C (mg/dL)	126.3±37.0	127.7±31.9	0.831
HDL- C (mg/dL)	51.7±12.5	51.8±11.8	0.965
Hemoglobin (g/dL)	12.8±1.5	13.0±1.4	0.425
Leukocytes (10^3^/mm^3^)	7.5±2.1	7.5±2.3	0.928
TSH	1.50 (0.23-8.3)	1.89 (0.4-8.5)	0.101
PAF episode (%)(n)	4.1 (8)	22.3 (23)	<0.001

BMI: Body mass index, MI: Myocardial infarction, LVEDD: Left ventricle end-diastolic diameter, LVESD: Left ventricle end-systolic diameter, PWd: Posterior wall end-diastolic thickness, ISDd: Interventricular septum end-diastolic diameter, LA: left atrium, BB: Beta blocking agent, ND-CCB: Non dihidropyridin calcium channel blocking agent, FPG: fasting plasma glucose, LDL-C: Low density lipoprotein cholesterol, HDL-C: High density lipoprotein cholesterol, TSH: tyroid situmulating hormone, PAF: Paroxysmal atrial fibrillation

**Table-II:** Patient characteristics according to the number of leads with fragmented QRS

	***Number of leads with FQRS***				
	***0-1 (n=198)***	***2-3 (n=87)***	***>3 (n=16)***	***p***
Gender (male) (%)	36.9	47.1	56.9	0.117
Age (years)	45.3±17.2^* β^	53.2±17.0^*^	55.2±15.8^ β^	0.001
BMI (kg/m^2^)	26.7±6.1	27.2±4.9	28.4±	0.511
Hypertension (%)	28.3	37.2	37.5	0.284
Diabetes mellitus (%)	10.6	18.8	25	0.074
Smoking (%)	25.3 (50)	23.3 (20)	18.8 (3)	0.812
Family history of CAD (%)	12.1 (24)^*β^	22.4 (19)*	31.3 (5)^ β^	0.023
Previus MI (%)	2 (4)	2.3 (2)	6.3 (1)	0.559
Ejection fraction (%)	63.4±4.1	62.1±5.6	60.7±10.9	0.127
LVEDD (mm)	42.6±4.6	44.0±4.6	43.3±6.3	0.204
LVESD (mm)	28.0±4.9	28.3±3.7	29.6±6.4	0.586
PWd (mm)	9.6±1.8	10.0±2.4	11.3±2.1	0.053
IVSd (mm)	9.4±2.2	10.1±1.9	10.9±1.5	0.059
LA (mm)	30.1±5.9*	33.1±6.1*	33.4±5.4	0.006
BB (%)	15.7*^ β^	27.6*	37.5^ β^	0.016
ND-CCB (%)	8.7	13.8	18.8	0.247
FPG (mg/dL)	92 (70-205)^*^	97 (73-221)^*^	97 (81-359)	0.015
Creatinin (mg/dL)	0.70 (0.47-4.15)	0.76 (0.49-2.69)	0.76 (0.52-1.36)	0.570
Total cholesterol(mg/dL)	193 (116-295)	196 (140-662)	225 (141-264)	0.580
Triglyceride (mg/dL)	95 (28-283)	119 (11-368)	92 (49-221)	0.248
LDL-C (mg/dL)	126.3±37.0	129.7±31.3	114.5±35.2	0.560
HDL- C (mg/dL)	51.7±12.4	51.2±11.8	57.5±10.4	0.624
Hemoglobin (g/dL)	12.8±1.5	13.0±1.4	13.4±1.5	0.477
Leukocytes (10^3^/mm^3^)	7.5±2.1	7.6±2.3	7.1±2.8	0.780
TSH	1.50 (0.23-8.30)^*^	2.05 (0.4-8.5)^*^	1.94 (1.02-2.36)	0.049
PAF episode (%)(n)	4.1 (8)^*^	14.9(13)^* β^	62.5 (10)^* β^	<0.001

^β ^significantly difference between into groups BMI: Body mass index, MI: Myocardial infarction, LVEDD: Left ventricle end-diastolic diameter, LVESD: Left ventricle end-systolic diameter, PWd: Posterior wall end-diastolic thickness, ISDd: Interventricular septum end-diastolic diameter, LA: left atrium, BB: Beta blocking agent, ND-CCB: Non dihidropyridin calcium channel blocking agent, FPG: fasting plasma glucose, LDL-C: Low density lipoprotein cholesterol, HDL-C: High density lipoprotein cholesterol, TSH: tyroid situmulating hormone, PAF: Paroxysmal atrial fibrillation.

**Table-III:** Univariate analyses for risk factors of atrial fibrillation episode

***Variable***	***OR (%95 CI)***	*** P ***
Age ≥65 years	3.62 (1.64-7.87)	0.001
LA ≥ 35 mm IVS ≥ 11 mm	3.55 (1.39-9.04) 4.75 (1.83-12.3)	0.008 0.001
Hypertension	3.80 (1.75-8.28)	0.001
Diabetes Mellitus	5.49 (2.40-12.54)	0.001
Family history	3.61 (1.59-8.20)	0.002
Male gender fQRS	0.52 (0.24-1.11) 6.79 (2.91-15.82)	0.094 0.001

OR: Odds ratio, CI: Confidence Interval, LA: left atrium, IVS: interventricular septum, fQRS: fragmented QRS

**Table-IV:** Independent predictors of atrial fibrillation episode

***Variable***	***OR (%95 CI)***	*** P ***
Hypertension	4.97 (1.31-18.76)	0.018
Diabetes Mellitus fQRS	2.20 (1.0-4.87) 9.69 (2.46-38.15)	0.024 0.001

OR: Odds ratio, CI: Confidence Interval, fQRS: fragmented QRS presence

There are many causes of AF and it is beyond the scope in this paper to discuss all of them but there are some changes in atrium that cause AF, regardless of the cause. Initially electrical remodeling of atrium occurs and then anatomic remodeling occurs which include; patchy fibrosis, excessive collagen deposition, fatty infiltration and apoptosis, finally resulting in an enlarged atrium.^18^ In our study we found that fQRS, HT, and DM were independently associated with the presence of PAF. Male predominance was observed in our study but it was not statistically significant. Age is also important for developing AF, although our study included relatively young patients, patient with fQRS were significantly older. LA diameter was found to be associated with AF development.^19^ In our study, although they were in normal range, LA diameter was significantly higher in patients with fQRS. This finding suggests that fQRS is not only associated with ventricular abnormalities, but it may also be associated with atrial structural abnormalities that need further investigation. Interestingly, others found that fQRS was associated with left ventricular mass index in patients with HT, which may also be associated with LA enlargement and AF development.^20^ In accordance with this finding IVS diameter was significantly greater in patients with fQRS. Thyroid disorders were also associated with AF, although we excluded the patients with thyroid diseases, TSH levels were higher in patients with fQRS, but this was not significant.

Many studies have demonstrated a relationship between various cardiovascular diseases and fQRS. The presence of fQRS was found to be associated with adverse cardiac events including; mortality, lethal arrhythmia, sudden cardiac death in these studies.^2^^-^^5^ Main causative mechanisms were explained by myocardial fibrosis that is expressed on the ECG as fQRS.^7^ Magnetic resonance imaging and myocardial perfusion studies showed the value of fQRS for detecting myocardial fibrosis ^7^. Except for fibrosis, myocardial ischemia may also cause fQRS due to altered myocardial depolarization of the myocardium^8^ Thirdly; some recent studies have demonstrated the role of inflammation for fQRS formation.^21^^,^^22^ So explaining the association between ventricular structural abnormalities, ventricular arrhythmias and fQRS is relatively easy. We could explain the association between fQRS and PAF as follows: fibrosis, ischemia, and/or inflammation alters both ventricular systolic and diastolic functions that results in elevated left ventricular end diastolic pressure (LVEDP) and this elevation in LVEDP reflects to the atrium and the atrium tries to adapt new condition and becomes vulnerable to arrhythmias.

***Study limitations: ***Firstly, this study involves a relatively small number of patients. Secondly, we didn’t evaluate the patients with magnetic resonance imaging (MRI) or myocardial perfusion scintigraphy to detect the myocardial abnormalities attributable to fQRS Thirdly, this is an observational study, further studies are needed to clarify whether the presence of fQRS is associated with clinical consequences of AF.

## CONCLUSION

In conclusion; fQRS is a sign of structural (fibrosis) and functional (ischemia, inflammation) myocardial abnormality and independently associated with presence of PAF in HM. Presence of fQRS seems to be a useful marker to help decision making for extended rhythm monitoring and managing patients to reduce the AF-related complications.
